# Cerebrospinal fluid and serum biomarkers of cerebral malaria mortality in Ghanaian children

**DOI:** 10.1186/1475-2875-6-147

**Published:** 2007-11-12

**Authors:** Henry B Armah, Nana O Wilson, Bismark Y Sarfo, Michael D Powell, Vincent C Bond, Winston Anderson, Andrew A Adjei, Richard K Gyasi, Yao Tettey, Edwin K Wiredu, Jon Eric Tongren, Venkatachalam Udhayakumar, Jonathan K Stiles

**Affiliations:** 1 Morehouse School of Medicine, Department of Microbiology, Biochemistry and Immunology, Atlanta, Georgia, USA; 2 University of Ghana Medical School, Department of Pathology, Accra, Ghana; 3 Noguchi Memorial Institute for Medical Research, Department of Parasitology, Legon, Ghana; 4 Howard University, Department of Biology, Washington DC, USA; 5 Centers for Disease Control and Prevention (CDC), National Center for Zoonotic, Vector-Borne and Enteric Diseases, Division of Parasitic Diseases, Malaria Branch, Atlanta, GA, USA

## Abstract

**Background:**

*Plasmodium falciparum *can cause a diffuse encephalopathy known as cerebral malaria (CM), a major contributor to malaria associated mortality. Despite treatment, mortality due to CM can be as high as 30% while 10% of survivors of the disease may experience short- and long-term neurological complications. The pathogenesis of CM and other forms of severe malaria is multi-factorial and appear to involve cytokine and chemokine homeostasis, inflammation and vascular injury/repair. Identification of prognostic markers that can predict CM severity will enable development of better intervention.

**Methods:**

Postmortem serum and cerebrospinal fluid (CSF) samples were obtained within 2–4 hours of death in Ghanaian children dying of CM, severe malarial anemia (SMA), and non-malarial (NM) causes. Serum and CSF levels of 36 different biomarkers (IL-1β, IL-1ra, IL-2, IL-4, IL-5, IL-6, IL-7, IL-8, IL-9, IL-10, IL-12 (p70), IL-13, IL-15, IL-17, Eotaxin, FGF basic protein, CRP, G-CSF, GM-CSF, IFN-γ, TNF-α, IP-10, MCP-1 (MCAF), MIP-1α, MIP-1β, RANTES, SDF-1α, CXCL11 (I-TAC), Fas-ligand [Fas-L], soluble Fas [sFas], sTNF-R1 (p55), sTNF-R2 (p75), MMP-9, TGF-β1, PDGF bb and VEGF) were measured and the results compared between the 3 groups.

**Results:**

After Bonferroni adjustment for other biomarkers, IP-10 was the only serum biomarker independently associated with CM mortality when compared to SMA and NM deaths. Eight CSF biomarkers (IL-1ra, IL-8, IP-10, PDGFbb, MIP-1β, Fas-L, sTNF-R1, and sTNF-R2) were significantly elevated in CM mortality group when compared to SMA and NM deaths. Additionally, CSF IP-10/PDGFbb median ratio was statistically significantly higher in the CM group compared to SMA and NM groups.

**Conclusion:**

The parasite-induced local cerebral dysregulation in the production of IP-10, 1L-8, MIP-1β, PDGFbb, IL-1ra, Fas-L, sTNF-R1, and sTNF-R2 may be involved in CM neuropathology, and their immunoassay may have potential utility in predicting mortality in CM.

## Background

Malaria is an important neglected disease and one of the most important global health problems, potentially affecting more than one third of the world's population. Cerebral malaria (CM) is a deadly complication of *Plasmodium falciparum *infection, associated with a 10–14% mortality rate and approximately 1–2 million annual deaths among young children predominantly in sub-Saharan Africa and Southeast Asia, yet its pathogenesis remains incompletely understood. In Ghana, malaria has a wide spectrum of presentations: from asymptomatic carriers to mild malaria to multifactorial severe disease, including CM and severe malarial anemia (SMA) [1-5].

CM, a clinically complex syndrome of coma and potentially reversible encephalopathy, is associated with increased levels of proinflammatory cytokines like tumor necrosis factor (TNF)-α, interferon (IFN)-γ and lymphotoxin [6,7], and increasingly recognized long-term sequelae in survivors [7-15]. Although the physiopathology of CM has been extensively investigated, the exact cellular and molecular basis of the neuropathology is still unclear. Recent studies have shown that mechanical blockage caused by sequestration of parasitized red blood cells (pRBCs), leukocytes and platelets [3,4,8-12], secretion of cytokines and chemokines [2,6,7,13], angiogenic failure [14,15], immune status and the genetic background of the host, and parasite factors [7,16] are involved in the pathogenesis of CM. However, it is generally accepted that two major factors are involved: (i) metabolic insufficiencies due to the sequestration of pRBCs, leukocytes and platelets within brain vessels via upregulated adhesion molecules [3,4,8-12], and (ii) immunological reactions with the local involvement of T cells and monocytes activated by *Plasmodium *antigens [7,11]. These two major mechanisms appear to act together under the control of cytokines [7], and chemokines [2] to exacerbate CM.

Postmortem data in humans and murine models of CM show that neuronal damage in brain tissue occurs in CM, although the parasites remain confined to the intravascular space (with no contact with neurons). This strongly suggests that the blood-brain barrier (BBB) is perturbed, and thus the BBB represents a key interface between the intraerythrocytic stages of the parasite and the human host. The functional and morphological evidence supports mild-to-moderate impairment of the BBB, but whether this is sufficient to cause neurological complications such as CM is inconclusive [16,17]. Mechanical blockage could occur from the ability of pRBC's to adhere to unparasitized erythrocytes and endothelial cells, and sequester in the deep cerebral microvasculature [3,4,8-12]. Parasite sequestration alone, however, cannot account for fatal CM pathogenesis as there is evidence that survivors of CM have the same degree of sequestration during comas as those succumbing to disease. It is evident that host immune factors play an important role in the pathogenesis of CM. [8,16]. Although parasite sequestration during severe malaria is central to pathogenesis of severe malaria, the role of cytokines, chemokines, apoptotic, and angiogenic factors in exacerbating disease severity remains unclear.

The balance between specific cytokines and chemokines produced in response to infection with *Plasmodium falciparum *is thought to play an important role in CM and other forms of severe malaria. Severe malaria has been associated with high TNF-α plasma levels in conjunction with increased production of IFN-γ and IL-1β [18,19] and decreased production of anti-inflammatory cytokines, notably IL-10 and TGF-β [1,19-22]. Pro-inflammatory Th1-type cytokines (eg. TNF-α, IFN-γ, interleukin IL-1β, and IL-6) are thought to be critical to the control of exoerythrocytic and erythrocytic *Plasmodium falciparum *infection [23,24], but their exaggerated production may also contribute to organ damage, particularly in the brain. It is widely accepted that anti-inflammatory Th2-type cytokines down-regulate Th1-derived cytokines. Th2-type cytokines, such as IL-10, has been shown to regulate Th1-cytokines and prevent CM in some animal models [18]. The regulation of TNF-α levels by IL-10 appears to contribute to the prevention of severe malarial anemia in humans [1,20,21]. However, the role that IL-10 plays may depend on its levels, since very high levels of IL-10 have been associated with severe malaria in humans [19] and some animal models [25]. Therefore, cytokines appear to maintain a delicate balance between the control of infection and contribution to disease in falciparum malaria infection. However, the expression of Th1 and Th2 cytokines in CSF, either from the peripheral circulation via the BBB or from neuronal immune cells (glia) has not been adequately addressed.

Chemokines, or chemoattractant cytokines, and their corresponding receptors have also been shown to mediate mobilization and coordination of immune responses to malaria. Chemokines have lympho-chemotactic activity and modulate many infectious and inflammatory diseases, including malaria. The recent demonstration of leukocyte sequestration, in addition to pRBC sequestration [3,4,8-12], within brain vessels in human CM suggests a more important role for leukocytes, including eosinophils, in CM immunopathology than previously thought. Thus, chemokines, including eotaxin, may play an important role in human CM by attracting leukocytes to sequestration sites. Chemokines are less well studied in severe malaria, but recent studies have associated severe malaria infection with increased production of chemokines of the C-C or β subfamily, including regulated upon activation, normal T cell expressed and secreted (RANTES), monocyte chemotactic protein (MCP)-1, macrophage inflammatory protein (MIP)-1α, MIP-1β, and IL-8 [2,13,26-28]. MCP-1, MIP-1α and MIP-1β are potent chemoattractants for monocytes to produce TNF-α and IL-6. IL-8 preferentially recruits neutrophils and plays an important role in inflammatory diseases. Recently, low levels of RANTES have been associated with severe malaria [26,27,29], and specifically associated with mortality in children with CM [26]. The low levels of RANTES in severe malaria have been associated with malaria-induced thrombocytopenia [26,29], given that platelets are a major reservoir of RANTES in the peripheral circulation. In contrast, increased mRNA and protein expression of RANTES and CCR5 was found in localized brain regions of children dying of CM [2]. Interferon inducible protein 10 (IP-10) is a member of the CXC or α subfamily of chemokines, and is induced in response to IFN-γ attracting activated Th1 cells [30]. IP-10 levels have been shown to increase in cultured intervillous blood mononuclear cells isolated from placenta's infected with malaria [31,32], although no studies have characterized IP-10 levels in human cerebral malaria. Hanum and colleagues recently demonstrated the induction of IP-10 expression in the brain of both CM-susceptible (C57BL/6) and CM-resistant (BALB/c) mice as early as 24 hours post-infection with *Plasmodium berghei *ANKA, and in KT-5 astrocyte cell line in vitro upon stimulation with a crude antigen of malaria parasites [33]. Currently, the role of chemokines in clinical severity and outcome of malaria, especially the development of CM and SMA in children remains poorly defined.

Angiogenic factors, long implicated as prognostic factors in cerebral ischemia or stroke [34], have been suggested to play a role in the petechial hemorrhages and BBB dysfunction associated with CM pathology [14,15]. Vascular endothelial growth factor (VEGF) stimulates endothelial cell growth and migration as well as enhancing vascular permeability. VEGF levels (more VEGF+ astrocytes) were higher in CM patients as compared with controls in a post-mortem immunohistology study of CM patients [15]. Platelets, that accumulate with pRBCs in the brain miscrovasculature in CM patients [10,12], are also implicated in CM pathology through TGF-β induced apoptosis in TNF activated human brain endothelial cells [35,36]. Platelet derived growth factor (PDGF) is another angiogenic factor that stimulates vascular growth, and has been implicated as a neuroprotective factor inducing regeneration of damaged axons and neuronal growth after ischemia [37]. These angiogenic factors play a dominant role in the recovery from stroke and may be applicable in CM due to their effect on the endothelium, which is central to CM pathology. These angiogenic factors most probably impact the regenerative potential of the parasite-induced BBB damage, rather than impacting neoangiogenesis, since CM is an acute neurological syndrome.

Parasite-induced apoptosis in the host may also mediate the severity of malaria. High levels of Fas-Ligand in sera of human [38,39], monkey [40], and mice [41] are associated with severity of malaria. Lymphocytes and macrophages express increased levels of Fas and Fas-Ligand during an acute *Plasmodium chabaudi *infection [40]. TNFR2-deficient mice are resistant to experimental cerebral malaria (ECM), Fas-deficient mice showed 50% reduction in ECM incidence, and TNFR1-deficient mice showed the least reduction in ECM incidence [42-46]. Lpr & Gld mice, deficient in Fas & Fas-Ligand, are protected from fatal ECM [47]. The presence of these apoptotic factors in CSF and serum, and their relevance in CM and CM-associated mortality has not been fully investigated. The rapid reversibility of the clinical symptoms of CM suggests that tissue necrosis is unlikely to occur [9,16,48], making apoptosis a more likely pathogenic mechanism.

Recently, new strategies including magnetic resonance imaging and ophthalmological evaluation of children with CM have been proposed as clinically useful predictors of CM severity, but their reliability is being evaluated. The study hypothesis was that parasite-induced dysregulation in the levels of inflammatory, apoptotic and angiogenic factors at the time of CM death would predict mortality risk of CM. The goal of this study was to identify factors that are tightly associated with CM mortality in Ghanaian children for further development as biomarkers of CM disease. The present study employed a high throughput multiplexed immunoassay to evaluate the predictive value of serum and CSF levels of key immunomodulators (inflammatory, apoptotic and angiogenic proteins) in determining mortality risk in severe malaria in Ghanaian children. We investigated the serum and CSF profiles of 36 different biomarkers (IL-1β, IL-1ra, IL-2, IL-4, IL-5, IL-6, IL-7, IL-8, IL-9, IL-10, IL-12 (p70), IL-13, IL-15, IL-17, Eotaxin, FGF basic protein, CRP, G-CSF, GM-CSF, IFN-γ, TNF-α, IP-10, MCP-1 (MCAF), MIP-1α, MIP-1β, RANTES, SDF-1α, CXCL11 (I-TAC), Fas-ligand [Fas-L], soluble Fas [sFas], sTNF-R1 (p55), sTNF-R2 (p75), MMP-9, TGF-β1, PDGF bb and VEGF) in order to identify the immune factors which influence progression to fatal outcomes associated with CM.

## Methods

### Case Selection

The post-mortem serum and CSF samples investigated were collected from children who died during the peak malaria season of 2005 (i.e. June-August), after being admitted to the Emergency Unit at the Department of Child Health, KorIe-Bu Teaching Hospital, Accra, Ghana. Only children for who detailed clinical and laboratory records and a clinically certified cause of death were available were included in the study. Samples were only collected only after written informed consent from parents or guardians of the deceased child. Nineteen (19) deceased children meeting the above inclusion criteria had serum and CSF samples removed at autopsy within 2–4 hours of death. Cadavers were moved immediately after consent had been given, from the Emergency Unit to the hospital's mortuary, for storage at 4°C. A full autopsy, with removal of serum and cerebrospinal fluid samples, was conducted on each consented case. At autopsy, blood samples of 2–5 mL were obtained by left ventricular aspiration, and CSF samples of 1–2 mL were obtained by lumbar or cistern puncture. Blood samples for serum testing were collected in a BD Vacutainer^® ^CPT Cell Preparation Tube [BD Diagnostics, New Jersey, USA], gently inverted 8–10 times, and then centrifuged for 20 minutes at 1500 rcf. These special tubes with polyester gel and density gradient liquid separated the blood into three distinct layers, namely serum, blood mononuclear cell and red blood cell layers. The separated serum and CSF were pipetted into aliquots and frozen at -70°C until testing was performed. Paired serum and CSF samples from all 19 children were available for testing.

The gross findings of the full autopsy and the examination of the brain, the observations on the brain smears and histological sections, and the data from the clinical and diagnostic-laboratory records of each subject were together used to classify the 19 cadavers into three illness groups. Nine (9) of the cadavers were categorized as cerebral malaria (CM), 5 as malaria complicated by severe anemia/severe malarial anemia (SMA), and 5 as non-malaria (NM) deaths. To be considered a case of CM while alive, a child had to fulfill the World Health Organization's definition of severe malaria [49], have a Blantyre coma score of ≤ 2; have a *Plasmodium falciparum *parasitemia, and have no other clinically evident cause of unconsciousness. At autopsy, the CM cases had a slaty-grey discoloration of the brain, white-matter petechial hemorrhages in the brain, and/or parasitized erythrocytes and/or malaria pigment in the cerebral microvasculature. While alive, the SMA cases had also clinically fulfilled the World Health Organization's definition of severe malaria [49], and also had a *Plasmodium falciparum *parasitemia, but were found to have no more than 5 g hemoglobin/dl and to remain conscious until shortly (< 2 hours) before their death. At autopsy, the brains of the SMA cases showed no slaty-gray discoloration, white-matter petechial hemorrhages, or parasitized erythrocytes and malaria pigment in the cerebral microvasculature, but all the internal organs of these cases had moderate to severe pallor. None of the NM cases (included as non-malarial controls) had been found clinically parasitemic, had normal biochemical and microbiological assessment of the CSF, and, at autopsy, none showed any slaty-grey discoloration of the brain, liver or spleen, or any white-matter petechial hemorrhages, or any parasitized erythrocytes, or any malaria pigment in his or her cerebral microvasculature, and no other gross or microscopic evidence of central nervous system (CNS) pathology. The study was approved by the Ethical and Protocol Review Committee of the University of Ghana Medical School (Accra, Ghana), and the Institutional Review Boards of both the Morehouse School of Medicine (Atlanta, GA) and the Noguchi Memorial Institute for Medical Research (Accra, Ghana).

### Multiplexed Microsphere Immunoassay

The 19 paired serum and CSF samples were evaluated simultaneously for 27 different circulating cytokines (IL-1β, IL-1ra, IL-2, IL-4, IL-5, IL-6, IL-7, IL-8, IL-9, IL-10, IL-12 (p70), IL-13, IL-15, IL-17, Eotaxin, FGF basic protein, G-CSF, GM-CSF, IFN-γ, IP-10, MCP-1 (MCAF), MIP-1α, MIP-1β, PDGF bb, RANTES, TNF-α and VEGF) using a commercially available multiplex colorimetric bead-based cytokine immunoassay coupled with the Luminex™ system (Austin, TX) and human-specific bead sets (BioRad, San Diego, CA), according to the manufacturer's instructions. The results were interpolated from 5-parameter-fit standard curves generated using the relevant recombinant human proteins (BioRad). Samples were tested at a 1:4 dilution.

### Enzyme-Linked Immunosorbent Assay

The 19 paired plasma and CSF samples were evaluated for the 9 other immune markers not available on the Multiplex-Luminex immunoassay system (TGF-β1 [latent and bioactive], sTNF-R2 (p75), sTNF-R1 (p55), sFas, Fas-L, SDF-1α, CXCL11 (I-TAC), MMP-9 and CRP) were measured by standard commercially available solid-phase sandwich ELISA kits, using human-specific primary and secondary antibodies (Biosource, R&D, and BD Pharmingen, San Diego, CA). The results were interpolated from 5-parameter-fit standard curves generated using the relevant recombinant human proteins (Biosource, R&D, and BD Pharmingen). Samples were tested at a 1:4 dilution.

### Statistical Analysis

Demographic variables were compared across the 3 study groups using analysis of variance (for continuous variables) and chi-squared testing (for categorical variables). Non-parametric test (Mann-Whitney rank sum test) was used for individual immune biomarker analysis between the 3 study comparison groups, while multivariate analyses (Least Squares with Bonferonni correction) was used to determine immune biomarker significance between the 3 groups after modeling and controlling for covariates (age, sex, and parasitemia). Correlations between immune biomarker levels were assessed by Spearman's rank correlation. Box plots representing medians with 25^th ^and 75^th ^percentiles, bars for 10^th ^and 90^th ^percentiles, and values outside the 10^th ^and 90^th ^percentiles of biomarker concentrations were plotted as points. Statistical significance for each biomarker was set at a two tailed P < 0.05, and Spearman's rank coefficients ρ > 0.25. The STATA™ (College Station, TX, USA) and SAS™ (Cary, NC, USA) Statistical Software were used to calculate statistics and plot graphs.

## Results

### Clinical and Diagnostic Characteristics of the Study Participants

The demographic, parasitological, and hematological characteristics of the 19 children (10 male, 9 female) investigated are summarized in Table 1. Children with SMA (5; 3 male, 2 female) were significantly (P = 0.021) younger than children with CM (9; 4 male, 5 female) and NM (5; 3 male, 2 female) (Table 1). Anemia (hemoglobin < 11 g/dl) was found among all the 3 study groups, but severe anemia (hemoglobin < 5 g/dl) was not found in the CM and NM groups. As expected, children with SMA had significantly (P < 0.01) lower hemoglobin levels than children with CM and NM, and CM children in turn had lower levels than children with NM (Table 1). All 5 children in the NM group were aparasitemic. The parasite density was significantly (P < 0.001) higher in children with SMA than CM (Table 1). Children with CM had significantly (P < 0.001) lower platelet count than children with SMA and NM, and SMA children in turn had lower levels than children with NM (Table 1).

**Table 1:** Demographic, parasitological, and hematological characteristics of the study participants

**CHARACTERISTIC**	**CM**	**SMA**	**NM**	**P value**
**No. of children**	9	5	5	
**Gender (male/female)**	4/5	3/2	3/2	0.249
**Age (months)**	61.2 (3.1)	14.6 (1.2)	79.0 (4.3)	0.021
**Parasite density (/μL)**	51,604 (9,468)	195,003 (23,613)	0	< 0.001
**Hemoglobin level (g/dL)**	7.2 (0.1)	3.7 (0.1)	7.9 (0.1)	< 0.01
**Platelet count (×10**^3^**/μL)**	170.8 (12.7)	288.4 (19.5)	330.7 (27.3)	< 0.001

CM, cerebral malaria; SMA, severe malarial anemia; NM, non-malaria; P < 0.05 considered statistically significant.

The 5 non-malaria (NM) cases investigated were made up of a case each of severe bronchopneumonia, severe gastroenteritis, abdominal tuberculosis, purulent bacterial peritonitis, and acute lymphoblastic leukemia. All 9 CM cases investigated had seizures, hypertonicity or posturing of limbs, and 4 CM cases had clinical and biochemical acidosis. None of the CM cases had hemoglobinuria, jaundice or renal failure. Although 4 of the 5 SMA cases investigated had hemoglobinuria, there was no clinical or biochemical evidence of renal failure. None of the SMA cases had clinical evidence of jaundice, an abnormal bleeding tendency, or meningitis or any other focus of infection. The CSF samples obtained from all 19 cases investigated were biochemically and microbiologically normal. No parasitized erythrocytes or sequestered mononuclear leukocytes were detected when the brain tissues of the 5 non-malaria cases studied were subjected to cytological and histological examination. Sequestered parasitized erythrocytes were seen, in the cytological and histological preparations, in brain microvessels of all the fatal malaria cases studied (CM and SMA), and sequestered mononuclear leukocytes (monocytes and lymphocytes) were seen in the preparations from 7 of the 9 CM cases but none of those from the SMA cases. In the cytological and histological preparations of the brain samples from the fatal malaria cases, the intra-erythrocytic malarial parasites were either nonpigmented or pigmented, and malaria pigment was found in intra-erythrocytic and intraleukocytic locations as well as lying free within the microvessels. The extent of sequestration and the distribution of malarial pigment in the cerebrum, cerebellum, brainstem, white matter and grey matter of each brain from the fatal malaria cases appeared identical.

### Serum Levels of Biomarkers in Children with CM, SMA, and NM

**Figure 1:**
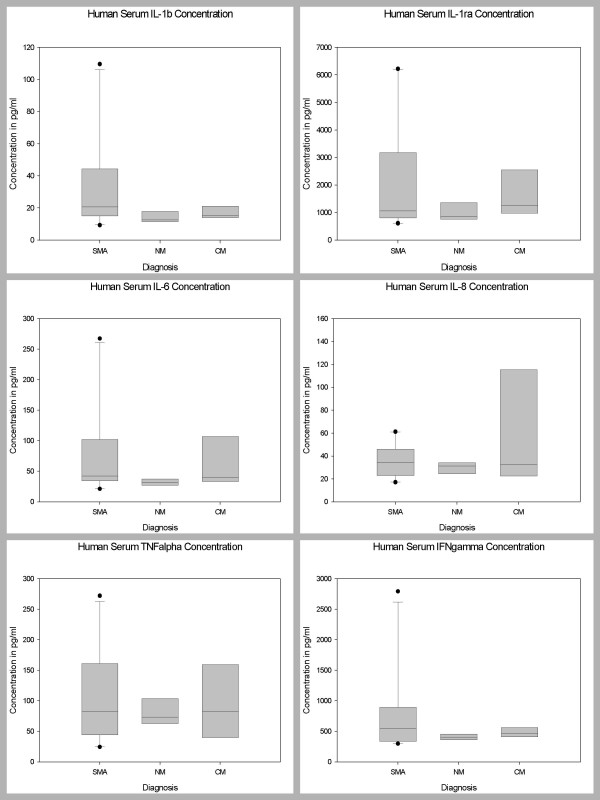
Postmortem serum biomarker levels of IL-1β, IL-1ra, IL-6, IL-8, TNF-α, and IFN-γ in children dying with CM, SMA, NM causes. CM, cerebral malaria; SMA, severe malarial anemia; NM, non-malaria. Box plots representing medians with 25^th ^and 75^th ^percentiles, bars for 10^th ^and 90^th ^percentiles, and points for outliers of biomarker concentrations. Only statistically significant P values after Bonferroni adjustment for the other biomarkers are shown.

**Figure 2:**
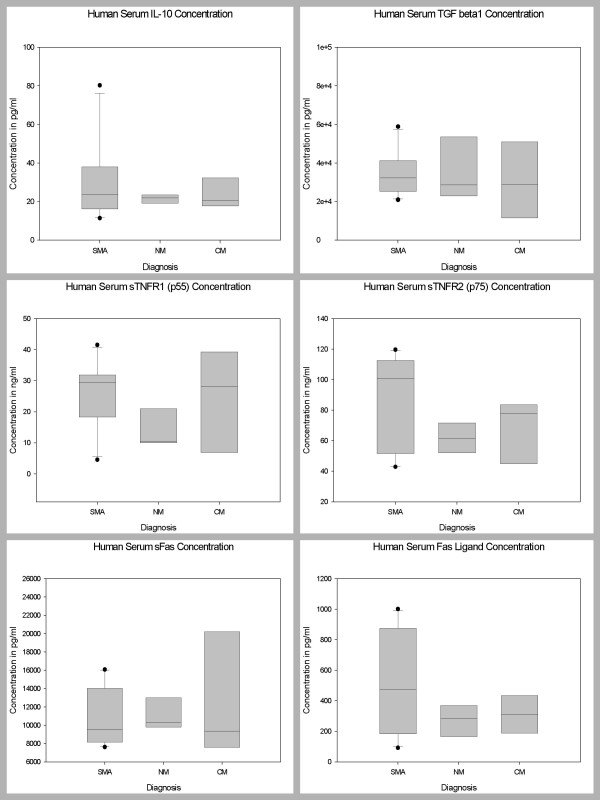
Postmortem serum biomarker levels of IL-10, TGF-βx, sTNF-R1, sTNF-R2, sFas, and Fas-L in children dying with CM, SMA, NM causes. CM, cerebral malaria; SMA, severe malarial anemia; NM, non-malaria. Box plots representing medians with 25^th ^and 75^th ^percentiles, bars for 10^th ^and 90^th ^percentiles, and points for outliers of biomarker concentrations. Only statistically significant P values after Bonferroni adjustment for the other biomarkers are shown.

**Figure 3:**
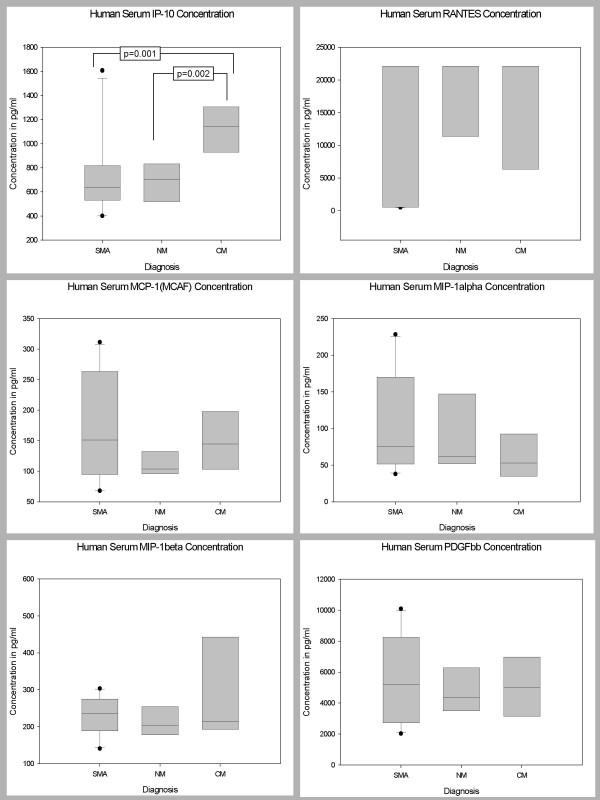
Postmortem serum biomarker levels of IP-10, RANTES, MCP-1, MIP-1α, MIP-1β, and PDGFbb in children dying with CM, SMA, NM causes. CM, cerebral malaria; SMA, severe malarial anemia; NM, non-malaria. Box plots representing medians with 25^th ^and 75^th ^percentiles, bars for 10^th ^and 90^th ^percentiles, and points for outliers of biomarker concentrations. Only statistically significant P values after Bonferroni adjustment for the other biomarkers are shown.

Pair wise comparisons were used to determine levels of significance of the differences between the serum biomarker levels of the 3 disease groups after controlling for age, sex and parasitemia. The serum levels of 35 biomarkers (IL-1β, IL-1ra, IL-2, IL-4, IL-5, IL-6, IL-7, IL-8, IL-9, IL-10, IL-12 (p70), IL-13, IL-15, IL-17, Eotaxin, FGF basic protein, CRP, G-CSF, GM-CSF, IFN-γ, TNF-α, MCP-1 (MCAF), MIP-1α, MIP-1β, RANTES, SDF-1α, CXCL11 (I-TAC), Fas-L, sFas, sTNF-R1 (p55), sTNF-R2 (p75), MMP-9, TGF-β1, PDGF bb and VEGF) demonstrated marginal changes, but did not show statistically significant differences between the 3 disease groups, after Bonferroni adjustment for the other biomarkers (Figures 1, 2, 3). However, serum level of IP-10 was independently predictive of CM mortality when compared to SMA and NM deaths. The serum level of IP-10 was significantly higher in children with CM compared with those children with SMA (P = 0.001) and NM (P = 0.002) (Figure 3).

### CSF Levels of Biomarkers in Children with CM, SMA, and NM

**Figure 4:**
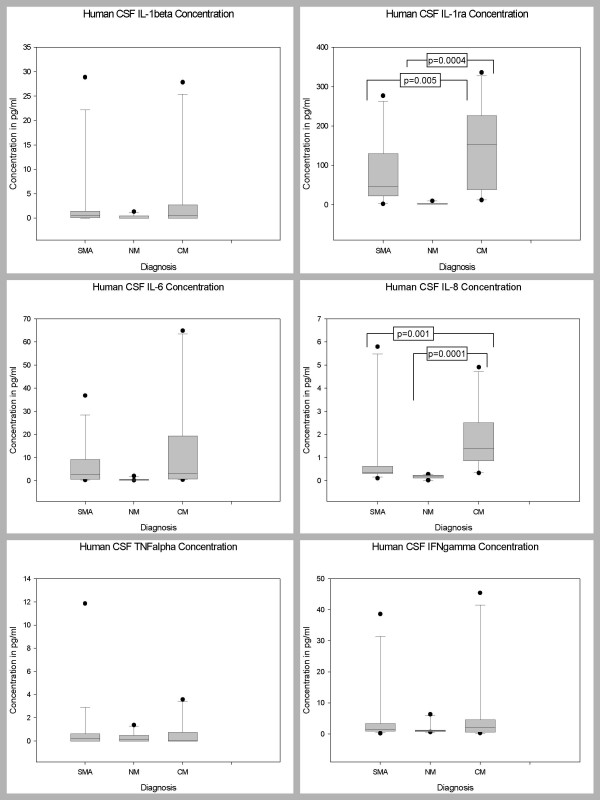
Postmortem CSF biomarker levels of IL-1β, IL-1ra, IL-6, IL-8, TNF-α, and IFN-γ in children dying with CM, SMA, NM causes. CM, cerebral malaria; SMA, severe malarial anemia; NM, non-malaria. Box plots representing medians with 25^th ^and 75^th ^percentiles, bars for 10^th ^and 90^th ^percentiles, and points for outliers of biomarker concentrations. Only statistically significant P values after Bonferroni adjustment for the other biomarkers are shown.

**Figure 5:**
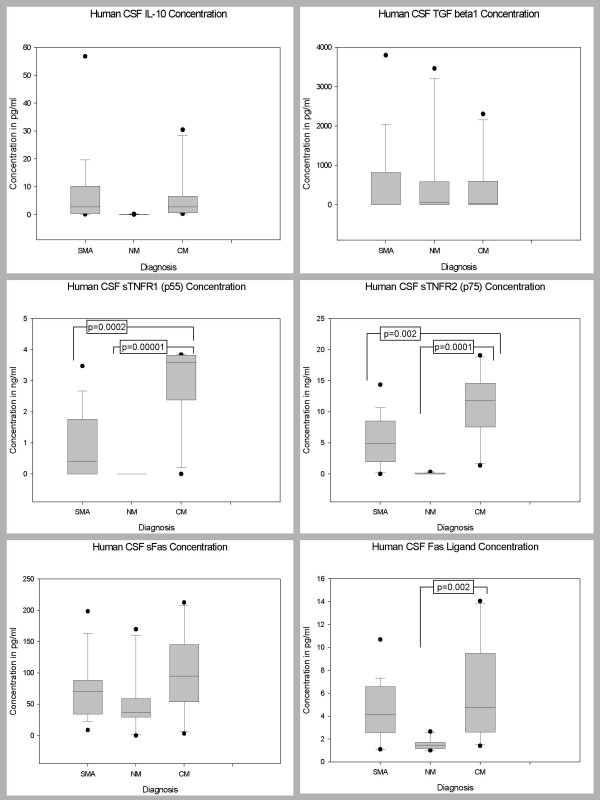
Postmortem CSF biomarker levels of IL-10, TGF-βx, sTNF-R1, sTNF-R2, sFas, and Fas-L in children dying with CM, SMA, NM causes. CM, cerebral malaria; SMA, severe malarial anemia; NM, non-malaria. Box plots representing medians with 25^th ^and 75^th ^percentiles, bars for 10^th ^and 90^th ^percentiles, and points for outliers of biomarker concentrations. Only statistically significant P values after Bonferroni adjustment for the other biomarkers are shown.

**Figure 6:**
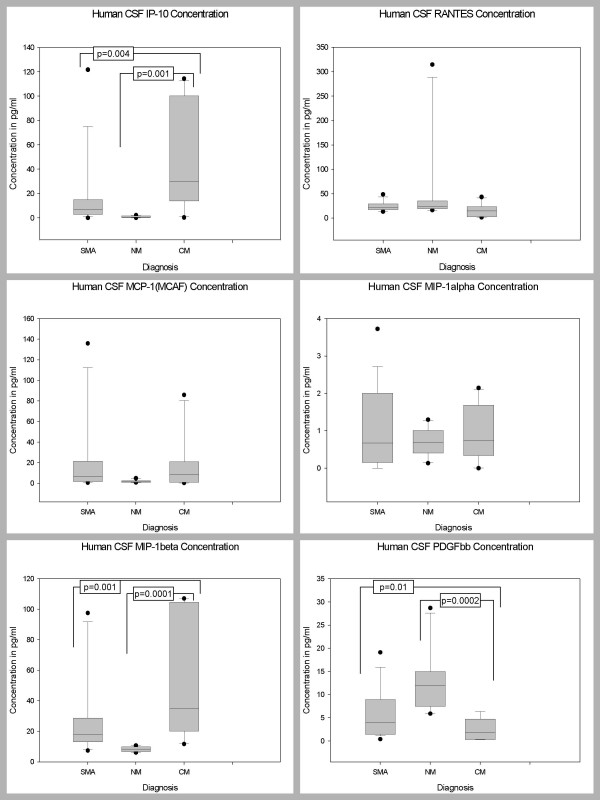
Postmortem CSF biomarker levels of IP-10, RANTES, MCP-1, MIP-1α, MIP-1β, and PDGFbb in children dying with CM, SMA, NM causes. CM, cerebral malaria; SMA, severe malarial anemia; NM, non-malaria. Box plots representing medians with 25^th ^and 75^th ^percentiles, bars for 10^th ^and 90^th ^percentiles, and points for outliers of biomarker concentrations. Only statistically significant P values after Bonferroni adjustment for the other biomarkers are shown.

Pair wise comparisons were used to determine levels of significance of the differences between the CSF biomarker levels of the 3 disease groups after controlling for age, sex and parasitemia (Table 2). The CSF levels of 27 biomarkers (IL-1β, IL-2, IL-4, IL-5, IL-6, IL-7, IL-9, IL-10, IL-12 (p70), IL-13, IL-15, IL-17, Eotaxin, FGF basic protein, CRP, G-CSF, GM-CSF, IFN-γ, TNF-α, MCP-1 (MCAF), MIP-1α, RANTES, SDF-1α, CXCL11 (I-TAC), MMP-9, TGF-β1, and VEGF) did not differ significantly between the three disease groups, after Bonferroni adjustment (Figures 4, 5, 6). The CSF levels of 9 biomarkers (IL-1ra, IL-8, IP-10, PDGFbb, MIP-1β, sFas, Fas-Ligand, sTNF-R1, and sTNF-R2) were independently predictive of CM mortality when compared to SMA and NM deaths (Figures 4, 5, 6). The CSF levels of IL-1ra, IL-8, IP-10, MIP-1β, sFas, Fas-Ligand, sTNF-R1, and sTNF-R2 were significantly higher in children with CM compared with those children with SMA and NM. On the contrary, PDGFbb was significantly lower in children with CM compared with those children with SMA and NM (Figures 4, 5, 6) (Table 2). These 9 biomarkers that were independently predictive of CM mortality can be grouped into four major categories such as cytokines (IL-8), cytokine receptors (IL-1ra, sTNF-R1, and sTNF-R2), chemokines (MIP-1β and IP-10), apoptotic (sFas and Fas-L), and angiogenic factors (PDGFbb).

**Table 2:** Comparison of Least Squares (Predicted) Means by Category, Controlled for Covariates (age, sex and parasitemia) For Biomarkers Showing Overall Statistically Significant Differences between the three Study Groups

**BIOMARKER**	**OVERALL P VALUE**	**P VALUE FOR PAIRED GROUPS**		
		
		**CM vs. NM**	**CM vs. SMA**	**SMA vs. NM**
**Serum IP-10**	0.005	0.002	0.001	NSS
**CSF IP-10**	0.005	0.001	0.004	NSS
**CSF IL-8**	0.0005	0.0001	0.001	NSS
**CSF MIP-1β**	0.0005	0.0001	0.001	NSS
**CSF PDGFbb**	0.008	0.0002	0.01	NSS
**CSF IL-1ra**	0.002	0.0004	0.005	NSS
**CSF Fas-L**	0.04	0.002	NSS	NSS
**CSF sTNF-R1**	0.0001	0.00001	0.0002	NSS
**CSF sTNF-R2**	0.001	0.0001	0.002	NSS

Statistically significant differences in biomarkers between the three disease groups determined by multivariate analyses [Comparison of Least Squares (Predicted) with Bonferonni correction] after modeling and controlling for covariates (age, sex, and parasitemia); P < 0.05 considered statistically significant; NSS, not statistically significant.

### Serum Biomarker Ratios in Children with CM, SMA, and NM

Serum pro-inflammatory or angiostatic to anti-inflammatory or angiogenic cytokine median ratios were determined and compared between the 3 disease groups (Table 3). Serum TNF-α/IL-10 median ratio was higher in CM group compared to the SMA and NM groups, but the difference was not significantly different (Table 3). Similarly, the serum TNF-α/IL-8, TNF-α/PDGFbb, IP-10/IL-10, IP-10/1L-8, and IP-10/PDGFbb median ratios were consistently higher in the CM group compared to the SMA and NM groups, but the differences were not statistically significant (Table 3).

**Table 3:** Comparison ofSelected Serum Biomarker Median Ratios between the three Study Groups

**PRO-INFLAMMATORY OR ANGIOSTATIC vs. ANTI-INFLAMMATORY OR ANGIOGENIC BIOMARKER MEDIAN RATIO**	**CM**	**SMA**	**NM**	**OVERALL P VALUE**
**TNF-α : IL-10**	4.11	3.22	3.18	NSS
**TNF-α : IL-8**	2.48	2.29	2.26	NSS
**TNF-α : PDGFbb**	1.64 × 10^-2^	1.54 × 10^-2^	1.56 × 10^-2^	NSS
**IP-10 : IL-10**	34.85	18.86	23.23	NSS
**IP-10 : IL-8**	2.31 × 10^-1^	1.27 × 10^-1^	1.63 × 10^-1^	NSS
**IP-10 : PDGFbb**	57.52	26.41	32.73	NSS

Median ratios of serum levels of pro-inflammatory/angiostatic versus anti-inflammatory/angiogenic biomarkers were compared between the 3 disease groups; P < 0.05 considered statistically significant; NSS, not statistically significant.

### CSF Biomarker Ratios in Children with CM, SMA, and NM

CSF pro-inflammatory or angiostatic to anti-inflammatory or angiogenic cytokine median ratios were determined and compared between the 3 disease groups (Table 4). CSF IP-10/PDGFbb median ratio was significantly higher in the CM group compared to the SMA and NM groups (Table 4). However, the CSF TNF-α/IL-10, TNF-α/IL-8, TNF-α/PDGFbb, IP-10/IL-10, and IP-10/1L-8 median ratios varied between the 3 disease groups, but the differences were not statistically significant (Table 4).

**Table 4:** Comparison of Selected CSF Biomarker Median Ratios between the three Study Groups

**PRO-INFLAMMATORY OR ANGIOSTATIC vs. ANTI-INFLAMMATORY OR ANGIOGENIC BIOMARKER MEDIAN RATIO**	**CM**	**SMA**	**NM**	**OVERALL P VALUE**
**TNF-α : IL-10**	3.33 × 10^-2^	7.51 × 10^-2^	2.04 × 10^-1^	NSS
**TNF-α : IL-8**	7.76 × 10^-2^	7.52 × 10^-1^	6.67 × 10^-1^	NSS
**TNF-α : PDGFbb**	5.05 × 10^-2^	7.56 × 10^-2^	1.53 × 10^-2^	NSS
**IP-10 : IL-10**	10.28	2.07	3.14	NSS
**IP-10 : IL-8**	23.08	20.12	10.29	NSS
**IP-10 : PDGFbb**	15.35	2.28	2.32 × 10^-2^	0.003

Median ratios of CSF levels of pro-inflammatory/angiostatic versus anti-inflammatory/angiogenic biomarkers were compared between the three disease groups; P < 0.05 considered statistically significant; NSS, not statistically significant.

### Correlation between Serum and CSF Levels of Biomarkers

The relationships of the serum and CSF levels of individual inflammatory markers with each other were examined using Spearman's rank correlational analyses. With the exception of IP-10, sTNF-R1 and sTNF-R2, there was no significant association between the serum and CSF levels of the other inflammatory markers studied (Spearman's ρ < 0.25; P > 0.05). There was a strong positive correlation between the serum and CSF levels of IP-10, sTNF-R1 and sTNF-R2 (Spearman's ρ = 0.58–0.82; all P < 0.0001) [data not shown].

### Correlation between Biomarker Levels and Clinical Characteristics

The relationships of individual inflammatory markers with each other, and with parasite density, hemoglobin level, and platelet count were examined using Spearman's rank correlational analyses. With the exception of IP-10 and MIP-1α, there was no significant association between the serum levels of the other inflammatory markers studied (Spearman's ρ < 0.25; P > 0.05). A moderately strong positive correlation was seen between the serum levels of IP-10 and MIP-1α (Spearman's ρ = 0.40; P = 0.001). With the exception of IL-1ra, IL-8, IP-10, PDGFbb, MIP-1β, sTNF-R1, and sTNF-R2, there was no significant association between the CSF levels of the other inflammatory markers studied (Spearman's ρ < 0.25; P > 0.05). The CSF level of PDGFbb correlated strongly and inversely with levels of IL-1ra, IL-8, IP-10, MIP-1β, sTNF-R1, and sTNF-R2 (Spearman's ρ = 0.61–0.76; all P < 0.0001). A moderately strong positive correlation was seen between the CSF levels of IL-1ra and sTNF-R1, IL-1ra and sTNF-R2, IL-1ra and IL-8, IL-1ra and 1P-10, IL-1ra and MIP-1β, and sTNF-R1 and sTNF-R2 (Spearman's ρ = 0.41–0.48; all P < 0.001). A weak positive correlation was seen between the CSF levels of IP-10 and MIP-1β, IP-10 and sTNF-R1, IP-10 and sTNF-R2, IP-10 and IL-8, MIP-1β and IL-8, MIP-1β and sTNF-R1, MIP-1β and sTNF-R2, IL-8 and sTNF-R1, and IL-8 and sTNF-R2 (Spearman's ρ = 0.26–0.37; all P < 0.05). Finally, there was no significant association between parasite density, hemoglobin level, and platelet count, and the serum and CSF levels of all the inflammatory markers studied (data not shown).

## Discussion

The present study examined a broad range of disease associated inflammatory mediators, including cytokines, chemokines, and markers of apoptosis and angiogenesis, in postmortem serum and CSF samples of children with CM, SMA, and NM. The study was conducted in an area of moderate *Plasmodium falciparum *transmission where all the life-threatening complications of malaria occur, namely, coma, severe anemia, and respiratory distress [1-5]. Although post-mortem studies have provided a wealth of detailed information they reflect, at best, pathology at a single time point after death in the most severely ill patients and may be potentially biased by post-mortem artifacts (agonal changes that may simulate disease-induced pathology). The concurrent studying of these inflammatory, apoptotic and angiogenic biomarkers in appropriate time-matched post-mortem controls from other disease causes helps place the results in context. Due to these limitations, further studies would be required to confirm the functional roles of these host factors in CM.

Cerebral malaria (CM) is a major life-threatening complication of *Plasmodium falciparum *infection in humans. The mechanisms underlying the fatal cerebral complications are still not fully understood. However, two predominant hypotheses are generally proposed to explain the neuropathology of CM, namely the sequestration and immunological hypotheses. The sequestration hypothetical model suggest that the adhesion of pRBCs to the cerebral vasculature leads to obstruction of the microcirculation, metabolic depletion, BBB breakdown, and alteration in brain function resulting in coma [16]. The immunological hypothetical model suggest that hyperimmune responses (originally evolved for the destruction of the parasite and protection of the host) and Th1/Th2 cytokine or chemokine dysregulation results in localized recruitment of immune effectors cells (T cells, monocytes, etc) and BBB impairment resulting in the development of cerebral complications [7]. However, recent studies indicate that parasite induced apoptosis and tissue degeneration, as well as angiogenic factors may be involved in the pathogenesis of CM [14,15,36,38]. Understanding the cytokine/chemokine cascade, parasite induced apoptotic pathways, and dysregulation of angiogenic factors in CM patients will elucidate the underlying pathogenesis and identify potential predictive prognostic biomarkers for CM mortality.

In the present study, evidence is provided indicating that the serum levels of various cytokines and chemokines are altered in children with CM compared to SMA and NM. The elevated serum level of IP-10 is particularly remarkable since it was the only independent predictor of CM mortality. Eight (8) CSF inflammatory biomarkers (IL-1ra, IL-8, IP-10, PDGFbb, MIP-1β, Fas-L, sTNF-R1, and sTNF-R2) were independently predictive of CM mortality, when compared to SMA and NM deaths. The significant increase in CSF levels of IL-1ra, IL-8, IP-10, MIP-1β, sTNF-R1, and sTNF-R2 in CM compared to SMA and NM suggests a critical role for the brain parenchymal expression of these biomarkers in CM pathogenesis and mortality. In the present study, both the serum and CSF levels of RANTES were not predictive of CM mortality, although low serum levels of RANTES have recently been associated with mortality in Ugandan children with CM [26].

TNF-R1 and TNF-R2 are key mediators of the classical extrinsic apoptotic pathway, as well as in inflammation. The increased expression of sTNF-R1 and sTNF-R2 in CSF of CM non-survivors when compared to SMA and NM suggests that parasite-induced apoptosis in host CNS is critical to CM pathogenesis and mortality. Recent studies in murine experimental CM have shown that the TNF receptor super family also plays a role in CM pathogenesis [42-47]. Mice deficient in TNF-R2 (TNF-R2-/-) and Fas (Fas-/-) survived significantly longer than wild type in experimental CM, and TNFR2-/- mice survived the longest in the absence of anti-malarial treatment [42,47]. Additionally, the serum levels of sTNF-R1 and sTNF-R2, which act as binding proteins for TNF, were elevated in patients with acute *Plasmodium falciparum *malaria compared to the levels in convalescent children and in healthy controls [38,39].

Platelet derived growth factor is a key factor that mediates vascular smooth muscle cell proliferation and serves a neuroprotective role by inducing regeneration of damaged axons and neuronal growth after ischemia [37]. In this study, a significant decline in PDGFbb production was independently predictive of CM mortality. Therefore, it seems that the down regulation of this angiogenic growth factor and upregulation of apoptotic factors in CM patients may be a result of parasite-induced damage or depletion of cells producing PDGF, a highly angiostatic microenvironment with high levels of proinflammatory cytokines/chemokines (notably IP-10), or even an unidentified parasite-derived factor that initiates/exacerbates the inflammatory and apoptotic cascades.

IFN-inducible protein of 10 kDa (IP-10) is a chemokine induced by IFN-γ and TNF-α. Although IP-10 was initially shown to have chemotactic activity for activated Th1 lymphocytes, there is growing evidence implicating this chemokine in both infectious and non-infectious causes of neuronal injury, dementia and inhibition of angiogenesis [50-54]. This is the first report demonstrating that significant elevation of serum and CSF levels of IP-10 is associated with CM mortality. Our finding suggests that IP-10 plays a major role in the CM immunopathology, and begs for further study in other endemic settings. Interestingly, *Plasmodium berghei *ANKA infection induced IP-10 and monocyte chemotactic protein (MCP)-1 gene expression in the brain of both CM-susceptible (C57BL/6) and CM-resistant (BALB/c) mice as early as 24 hours post-infection [33]. Additionally, the expression of IP-10 and MCP-1 genes in KT-5, an astrocyte cell line, was induced in vitro upon stimulation with a crude antigen of malaria parasites, suggesting astrocytes as the potential cellular source of cytokine and chemokine expression in brain parenchyma in response to plasmodial infection [33]. Therefore, in response to plasmodial infection, the cells that produce these inflammatory mediators may be different in the brain (microglia and astrocytes) and peripheral circulation (platelets, monocytes, and lymphocytes), and their effects may also differ in the two areas.

This study has revealed new associations, underlying pathogenic events, between different biomarkers and CM mortality in Ghanaian children that may be applicable to other malaria endemic populations. The most important finding demonstrates the association between the elevation of serum and CSF factors involved in the classical extrinsic apoptotic pathways (such as IP-10, TNF-α-sTNF-R1-sTNF-R2 and Fas-L) and the reduction of growth factors that confer endothelial and neuronal cell protection (such as PDGF) with CM mortality. We propose the following hypothesis to explain our observations. It appears that both inflammatory and apoptotic mechanisms may be triggered locally in the human brain during CM that result in the damage of the constituent cells of the BBB (glial cells, astrocytes, and endothelial cells) and possibly neurons. Additionally, this localized BBB damage may be further exacerbated by the significantly decreased levels of neuroprotective angiogenic growth factors (such PDGF), induced by the angiostatic effects of the elevated local CSF levels of IP-10, ultimately resulting in death.

Furthermore, we propose that TNF-α and other proinflammatory factors which are activated following the release of malaria antigens after schizont rupture may induce the local production of IP-10 by the constituent cells of the BBB (glial cells, astrocytes, and endothelial cells) [33]. Subsequently, IP-10 in concert with TNF-α may induce apoptosis of endothelial cells leading to BBB breakdown. Additionally, activated circulating immunomodulator cells (T cells, monocytes, etc) attracted to the BBB by IP-10, may also play a pathogenic role in this process. The significantly decreased production of PDGF may further inhibit angiogenesis and negatively impact the regeneration of damaged endothelial cells and blood capillaries at the BBB. Recently, elevated CSF level of IP-10 has been demonstrated in viral meningitis [54]. Elevated CSF level of IP-10 has been shown to be significantly correlated with the neuropsychiatric impairment in HIV-associated dementia [55]. Furthermore, mouse studies have demonstrated that the HIV-1 virus encoded protein gp120 directly activates astrocytes to produce IP-10 using a novel mechanism independent of IFN-γ and STAT-1 pathway of IP-10 induction [52]. Therefore, elevated serum and CSF levels of IP-10 may be an important pathogenic factor in CM neuropathology, as observed in other infectious disease models. Most CM deaths occur within 24 hours of admission before antimalarials have had time to kill the parasites [9,16,48], hence new interventions that address pathophysiological processes causing these early deaths is a public health priority, in addition to addressing the public-health problems resulting in delayed presentation to hospital and ensuring children receive prompt and appropriate resuscitation. Thus, this study provides new insights into the processes leading to cerebral malaria and mortality associated with it.

## Conclusion

This study has demonstrated an association between CM associated mortality with elevated serum and CSF levels of apoptotic factors (IP-10, IL-1ra, sTNFR1, sTNFR2, sFas) and reduced serum and CSF levels of neuroprotective angiogenic growth factors (PDGFbb). The observations support recent reports that implicate parasite-induced apoptosis and angiogenic factors in CM neuropathology. Further studies in other endemic areas to confirm these findings are necessary.

## Abbreviations

BBB, Blood-Brain Barrier;

CM, cerebral malaria;

CNS, central nervous system;

CRP, C-reactive protein;

CSF, cerebrospinal fluid;

ECM, experimental cerebral malaria;

FGF, fibroblast growth factor;

G-CSF, granulocyte colony stimulating factor;

GM-CSF, granulocyte-monocyte colony stimulating factor;

IFN, Interferon;

IL, Interleukin;

IP, Interferon inducible protein 10;

MCP, monocyte chemotactic protein;

MIP, macrophage inflammatory protein;

MMP, matrix metalloproteinase;

NM, non-malaria;

PDGF, platelet derived growth factor;

pRBC, parasitized red blood cell;

RANTES, regulated upon activation, normal T cell expressed and secreted;

SDF, stromal differentiation factor;

SMA, severe malarial anemia;

TGF, transforming growth factor;

TNF, Tumor Necrosis Factor;

VEGF, vascular endothelial growth factor;

## Competing interests

The author(s) declare that they have no competing interests.

## Authors' contributions

**HBA **performed the autopsies and sample collection, immunoassays, data analysis and drafting of the manuscript.**NOW **and **BYS **participated in the performance of immunoassay, data analysis and drafting of the manuscript. **MDP, VCB, JET **and **VU, **participated in the performance of proteomics analysis.**WA, AAA, RKG, YT **and **EKW **participated in the design and coordination of the study, and supervised the autopsies and sample collection. **JKS **conceived of the study, participated in its design and coordination, and revised the manuscript for important intellectual content. All authors read and approved the final manuscript.
